# Bladder augmentation: Review of the literature and recent advances

**DOI:** 10.4103/0970-1591.36721

**Published:** 2007

**Authors:** Serhat Gurocak, Jody Nuininga, Iyimser Ure, Robert P. E. De Gier, Mustafa Ozgur Tan, Wouter Feitz

**Affiliations:** Gazi University School of Medicine, Department of Urology, Section of Pediatric Urology, Ankara, Turkey; * Pediatric Urology Centre Nijmegen, Department of Urology, Radboud University Nijmegen Medical Centre, The Netherlands

**Keywords:** Bladder augmentation, children, enterocystoplasty, future perspectives, large bowel, review, small bowel, stomach

## Abstract

Bladder augmentation is an important tool in the management of children requiring reconstructions for urinary incontinence or preserving of the upper urinary tract in congenital malformations. We reviewed the literature and evaluated the long-term results of enterocystoplasty in the pediatric age group and summarized techniques, experimental options and future perspectives for the treatment of these patients. For this purpose, a directed Medline literature review for the assessment of enterocystoplasty was performed. Information gained from these data was reviewed and new perspectives were summarized. The ideal gastrointestinal (GI) segment for enterocystoplasty remains controversial. The use of GI segments for enterocystoplasty is associated with different short and long-term complications. The results of different centers reported in the literature concerning urological complications after enterocystoplasty are difficult to compare because of the non-comparable aspects and different items included by different authors. On the other hand, there are more and more case reports about cancer arising from bowel segments used for bladder augmentation in recent publications.

Although bladder reconstruction with GI segments can be associated with multiple complications, such as metabolic disorders, calculus formation, mucus production, enteric fistulas and potential for malignancy, enterocystoplasty is unfortunately still the gold standard. However, there is an urgent need for the development of alternative tissues for bladder augmentation.

Bladder augmentation (BA) with GI tissue is an important tool in the armamentarium of the urologist in the management of children requiring reconstruction for urinary incontinence, preserving the upper urinary tract and in reconstructions for severe congenital malformations. The goal of BA is to create a reservoir with an adequate functional capacity with a low end-filling pressure. By achieving this, the low intravesical pressure will not interfere with ureteral delivery of the urine to the bladder and preserve the upper urinary tract from high pressure damage by vesico-ureteral reflux.

To the best of our knowledge, Simon first reported the use of tissues other than urothelial tissues to achieve this goal 150 years ago.[1] He clinically applied a ureterosigmoidostomy to a child with bladder exstrophy. Mikulicz was the first who described the use of small intestine to augment the bladder in 1899.[2] Yeates described the use of detubularized small intestine and this method has since been shown to be an effective method of providing a compliant reservoir.[3] However, this was not a general practice at that time, because enterocystoplasty was known to result in urinary retention. So, most pediatric patients were generally diverted from augmentation procedures due to the retention problems, until Lapides *et al*., proved the efficiency of clean intermittent catheterization (CIC) to empty the bladder.[4] After this important cornerstone, augmentation cystoplasty with various intestinal segments grew rapidly and changed the management of pediatric and adult patients with abnormal bladder function. The current techniques for pediatric BA are cystoplasty with stomach,[5–6] large bowel,[7–8] small bowel,[9–13] ileocaecal,[14] ureter[15] and engineered tissue[16] with or without urinary continence procedures. Neuropathic dysfunction (myelomeningocele) and bladder exstrophy are the main underlying conditions for pediatric patients requiring an enterocystoplasty. Major postoperative complications like metabolic disorders, hematuria syndrome, calculus formation, mucus production, enteric fistulas, bladder rupture, intestinal obstruction and the potential for developing a malignancy are associated with the use of GI tissues in the bladder.[17–28] Although bladder reconstruction with GI is associated with several complications, as well as the morbidity of harvesting the segment of bowel, enterocystoplasty is still considered to be the best of all alternatives. The aim of this review is to discuss the details of BA which include the indications, type of material, the long-term outcome, alternative options, experimental and future options.

## AUGMENTATION WITH GASTRIC TISSUE, SMALL AND LARGE BOWEL

### Technique

Gastrointestinal segments from the stomach to the sigmoid have been used for an enterocystoplasty. Only the jejunum quickly fell into disrepute because of salt losses and acidosis. Augmentation enterocystoplasty is performed using a detubularized segment of ileum (10), caecum (14) or sigmoid (7).

### Gastric tissue

Gastric tissue has been used as donor tissue for bladder augmentation for over 30 years. It was popularized by Adams *et al*., in 1984 for use in children who had no other bowel available (e.g. cloacal exstrophy and after pelvic irradiation) or in patients who could not tolerate metabolic acidosis from resorption of chloride.[5] He noted an 85% success rate in their study group. They found after gastrocystoplasty comparable results to other bowel segments. Although gastric augmentation usually results in an improvement in bladder volume and compliance, the stomach segment continues to act as a gastric secreting organ, which may predispose to hyperchloremic hypokalemic metabolic alkalosis, hematuria, dysuria syndrome and gastrinemia.[6] Chronic electrolyte imbalance may also lead to abnormalities in calcium homeostasis, resulting in bone demineralization.[27] This might compromise growth and development of children.[28] Mingin *et al*., found a complication rate of 36% after a mean follow-up of six years.[6] They conclude, considering the major metabolic and physiological complications of gastrocystoplasty, that the stomach should be limited to patients who have undergone bowel irradiation and those at risk for short bowel syndrome, as in cloacal exstrophy. This was also mentioned by Adams before.[5]

### Small and large intestine

Many urologists hold bladder augmentation with small intestine as the procedure of choice when conservative managements fails. In 1982 Goodwin[9] first described this procedure, which was popularized later by Mundy and Stephenson.[11] Mundy *et al*., reported a 90% success rate in augmentation cystoplasty performed in 40 patients with neuropathic bladder dysfunction with a mean follow-up of one year. Several other reports have consequently confirmed the high success rate to achieve a low-pressure, high-capacity urinary reservoir. Krishna *et al*., studied 39 children with spina bifida and reported 91.7% reduction in upper tract dilatation, which was distended preoperatively.[12] Riedmiller *et al*., used ileum for bladder augmentation and accomplished large compliant bladders with moderate mucus production.[13] In addition, they found a low complication rate with this technique.

On the other hand, augmentation procedures with the integration of bowel segments into the urinary tract have numerous disadvantages. It is clear that intestinal segments used in the urinary tract retain their native function of secreting or reabsorbing salt and water from the urine. This can result in a multitude of metabolic disturbances.[25,26] Resection of large parts of the distal small bowel can result in chronic B12 and folate deficiency.[27] Long-term follow-up of hematological laboratory tests are needed. The use of bowel in the urinary tract has been associated with chronic bladder infections and a tendency for bladder stone formation.[22] In addition, it must be kept in mind that enterocystoplasty is a major intraperitoneal surgery with various complications and patients should be counseled about the significant probability of the need for CIC postoperatively ranging from 15%–75%.[28] The possible surgical complications of these procedures are anastomotic leakage, stricture, intestinal obstruction or bladder rupture.[17–21]

The ideal bowel segment for enterocystoplasty remains controversial. Kilic *et al*.,[29] conclude that ileocystoplasty is the procedure of choice to achieve a low-pressure, high-capacity urinary reservoir. In addition, they found a low complication rate in this technique. Others found more complications after ileocystoplasty in comparison to sigmoidocystoplasty.[17] We found a comparable complication rate of 7/14 sigmoidocystoplasty patients in comparison to 13/27 in the ileocystoplasty group by Nuininga *et al*.[30] The ileum is used most frequently, preferably as an ileal conduit, possibly due to the accumulated experience with this technique because of other pathological conditions.

The results of different centers reported in the literature concerning urological complications after enterocystoplasty are difficult to compare because of the non-comparable aspects and different items included by different authors (e.g. with or without including urinary tract infections, pyelonephritis, renal transplantation). Many studies report on pediatric and adult patients together with different follow-up periods. Sometimes only a brief referral is given about these complications in publications about enterocystoplasties.

### Bladder perforation

Rupture of the augmented bladder is an uncommon but one of the most serious and life-threatening complications of enterocystoplasty. Factors that are thought to contribute to the risk of perforation include choice of bowel segment, high bladder pressures, configuration of the bowel segment, traumatic catheterization, chronic infection and ischemic necrosis of the intestinal segment. Several studies evaluated these risk factors for spontaneous bladder perforation after augmentation cystoplasty. DeFoor *et al*., found a low incidence of spontaneous bladder perforation of 5% in 107 children.[31] They explained this by the large number of patients with gastrocystoplasty, as well as their strict adherence to a postoperative incremental catheterization program. All patients postoperatively got temporary cystostomy tubes for four to six weeks and then patients started intermittent catheterization on a regular or incremental schedule according to surgeon preference. Metcalfe *et al*., reported one of the largest series of bladder augmentations and found a perforation rate of 8,6% in 500 enterocystoplasty patients.[32] This large and comprehensive series of patients gives valuable insight into this serious complication. A significant increased risk of perforation was observed with the use of sigmoid colon and bladder neck surgery. A decreased risk was associated with the presence of a continent catheterizable channel. Reservoir perforation must be considered in patients with sudden lower abdominal pain or peritonitis associated with low-grade fever.

### Stone formation

The incidence of calculi in augmented bladders is high, but varies between published studies. In most, it is observed in 2–18% of all patients. In one report of children, an extraordinary 52.5% had formed a stone at a mean follow-up of four years.[33] In enterocystoplasties one important factor in the formation of bladder calculi is the production of mucus. The mucus may probably enhance stone formation directly by acting as a heterogeneous nucleator or indirectly by facilitating bacterial growth. This was confirmed by the fact that the frequency of stone formation seems to depend on the frequency with which the patients irrigate the bladder free of mucus.[34] But others found no correlation between the frequency of bladder irrigations and stone formation.[22,35] They found urinary tract infection an independent risk factor for stone formation. The care should include clear emphasis on the role of treating symptomatic urinary tract infections. Another risk factor for stone formation is foreign bodies, such as staples, in the reservoir.[36] This increases the risk of stone formation from 13% to 43%.

### Neoplasia

Cancer following augmentation cystoplasty is a recognized risk factor. In recent publications there are more and more case reports about cancer arising from bowel segments used for bladder augmentation. The majority of tumors have been adenocarcinoma but transitional cell carcinoma (TCC) has also been reported. The majority of reported cases of post augmentation malignancy have occurred in adults with other multiple potential risk factors.[23] It is difficult to determine the exact independent risk associated with bladder augmentation. Recently, Soergel reported three cases (1.2%) of transitional cell carcinoma (TCC) following augmentation cystoplasty in a unique population of 260 augmentations with at least 10 years of follow-up with no additional risk factors for bladder cancer.[24] No patient had a history of smoking exposure or other known risk factors for bladder cancer. Mean time from bladder augmentation to TCC was 19 years. This study supports the hypothesis that bladder augmentation appears to be an independent risk factor for TCC. They recommend endoscopic surveillance of all patients with a history of bladder augmentation beginning 10 years after initial surgery.

## ALTERNATIVE OPTIONS

### Minimally invasive options

#### Oxybutinin and CIC

Many investigators have attempted to use alternative materials and tissues for replacement of the gastrointestinal tissue. An alternative minimal invasive treatment might be conservative management with anticholinergic therapy and clean intermittent catheterization (CIC). This conservative management was performed by many authors and recently by Dik *et al*.,[37,38] in a prospective study. Fifteen spina bifida patients were all on a regime of CIC and anticholinergic therapy shortly after birth. They found that detrusor overactivity recurred immediately after the cessation of the oxybutinin and concluded that no long-lasting suppressive effect should be expected from these drugs because of a primary neuropathic origin.

#### Botulinum-A toxin

Another alternative therapeutic option might be the use of Botulinum-A toxin injection to the detrusor in patients with a neuropathic bladder. Schurch *et al*., were the first to apply the toxin into the neuropathic overactive detrusor and achieved increased bladder capacity and decreased pressures.[39] Riccabona *et al*., operated 15 patients with myelomeningocele.[40] All patients were injected with 10U/kg of botulinum toxin-A at 25–40 sites in the detrusor. The urodynamic evaluation of the efficacy and durability of Botulinum toxin-A was done at three, nine and 12 months. They found significant improvement in bladder compliance, bladder capacity and decline in maximum bladder pressure at nine months. But at 12 months the encouraging results reverted to preoperative results. Although this study showed the safety of this minimal invasive operation, the need for recurrent treatment each nine months is a big disadvantage. But this might be better than the several complications with augments. Future studies including randomization, controls and long-term follow-up are mandatory before this treatment in patients with a neuropathic bladder is taken into daily clinical use.

### Invasive options

#### Ureterocystoplasty

Ureterocystoplasty might be indicated in patients who have poor compliance. In a typical case, function of one kidney in a patient is affected and the affected kidney has poor renal function with a massively dilated collecting system. In this procedure, the dilated ureter is used for bladder augmentation and therefore it avoids the complications associated with intestinal mucosa.[41] Perovic *et al*. have performed ureterocystoplasty in 16 patients and they used the distal part of the megaureter for bladder augmentation and implanted the proximal part into the bladder using extravesical tunneling ureteroneocystostomy. With this technique, they provided an increased bladder capacity between 296 to 442ml (mean 371) and an increase in bladder compliance without any further worsening of renal function.[42] Nahas *et al*., assessed clinical and surgical results in renal transplantation candidates with voiding dysfunction and end stage renal disease who underwent bladder augmentation with ureterocystoplasty technique. They analyzed eight patients and they found that the bladder capacity was increased and intravesical pressure was decreased in these patients after the surgery with a mean serum creatinine of 1.65mg/dl. This study shows that ureterocystoplasty can also be used with confidence in end stage renal disease because it combines the benefits common to all enterocystoplasties without adding any complications.[43] In a larger study, Husmann *et al*., found similar results such as increased bladder capacity and compliance in 64 patients who underwent ureterocystoplasty.[44] Dewan *et al*., described the use of ureterocystoplasty in bladder extrophy[45] and Clento Jr *et al*., performed laparoscopically assisted ureterocystoplasty safely in five patients.[46] These studies show us that this technique is a safe and reliable procedure.

#### Demucosalized enterocycstoplasty

An alternative technique for providing urothelium-lined bladder to avoid the complications of bowel mucosa contact with urine including malignancy, mucus and stone formation, metabolic acidosis and reduced linear growth in children, is augmentation demucosalized enterocystoplasty. In this technique denuded intestinal muscle is used for bladder augmentation.[47] Lima *et al*., presented long-term results on the use of demucosalized intestine for reconstructive surgery of the bladder which was performed in 129 bladder augmentations on 123 patients with 10 years of follow-up. They used sigmoid in 104 cases and ileum in 25 cases. They found a 329% increase in bladder capacity and sevenfold increase in compliance after surgery. Thirteen cases were considered as failure and they performed reaugmentation for 11 of these patients. These data shows that demucosalized intestinal flaps are appropriate for bladder reconstruction for the same indications as total flaps.[48] In another study by Lima *et al*., demucosalized ileum was used for augmentation of the bladder in 11 patients and a Foley catheter with inflated balloon was used for dissection of the mucosa from the muscle. They provided a significant increase in bladder compliance in all patients. They supported this study with an animal model and good-shaped bladders were obtained in these animal models.[49] In addition, Lima *et al*., performed another animal model with 12 female dogs which underwent total cystectomy and bladder replacement by neobladder composed of demucosalized ileal segment and they divided their study population into two groups. In the first group, an intravesical silicone modeler was used for preventing graft retraction in contrast to the second group. They found a significant difference in bladder capacity between the groups after surgery. The bladder capacity was significantly larger in Group I.[50] In another animal study by Vates *et al*., they declared that preservation of the submucosa of the demucosalized colonic segment is essential to prevent fibrosis and a balloon stent is crucial to prevent graft contraction. Treatment of the demucosalized segment with protamine sulfate and urea results in better urothelial expansion and less colonic mucosal regrowth.[51] The main problem of this technique is the tissue contracture frequently encountered in demucosalized segments. This phenomenon might show us the importance of vascular supply contribution of the colonic mucosa to all segments of the intestine. Animal models like silicone balloons which are mainly maneuvers to prevent contraction might be intractable in human being.

#### Autoaugmentation

A number of strategies have been devised to avoid the use of bowel in the urinary tract. Cartwright and Snow described the first autoaugmentation technique in dogs in 1989. Although the follow-up time was short, they reported in their first clinical study a subjective success of good in three (43%) and excellent in four (57%) of the seven patients. They declared that autoaugmentation could be an alternative to enterocystoplasty in a select group of patients whose compliance is poor based on abnormal pressures. These same workers declared in a subsequent report with a longer follow-up period that 80% of patients were continent although a significant increase in bladder capacity was not observed.[52] Although the results of the aforementioned studies were promising, doubts remain about the reliability and long-term efficacy of this technique. Marte *et al*., reviewed their autoaugmentation results in myelodysplastic children and found that this procedure failed in seven of 11 patients after 6.6 years.[53] They concluded that patient selection seemed to be crucial for the success of autoaugmentation. Macneily *et al*., found that clinical outcome did not appear to be durable.[54] Four of 17 patients (23,5%) required enterocystoplasty and 12 (71%) patients were considered to be clinical failures due to upper tract deterioration and ongoing incontinence. The disadvantage of autoaugmentation is limitation in the postoperative bladder expansion when compared to conventional enterocystoplasty. In addition, Gurocak *et al*.,[55] reviewed autoaugmentations and concluded that achievement of better compliance after autoaugmentation procedures seems to be less pronounced and of shorter duration than that of conventional enterocystoplasty. On the other hand, the low morbidity and lack of side-effects of bowel integration into the urinary tract are the definite advantages of this technique. It was concluded that it is the responsibility of the physician to determine the balance between the limited efficacies of the procedures versus the definite advantages.

### Experimental options

During the last few decades many investigators have directed their attention to alternatives for bladder augmentation or reconstruction. Studies in animals with biocompatible and biodegradable biomatrices are promising.[56,57] Currently, the two most common tissue engineering techniques for bladder reconstruction are regeneration with unseeded or seeded biodegradable biomatrices. The unseeded technique, involves reconstruction of the bladder with a biocompatible and biodegradable matrix. The success of this repair depends on the natural regeneration capacity of the bladder. The seeded technique involves *in vitro* cell culture techniques to create a biomatrix with primary cultured epithelium on one side and smooth muscle cells on the other side. This *in vitro* prepared ‘bladder wall’ is used in the host for bladder repair. Reconstruction of the urinary tract using the principles of tissue engineering now face the challenge of determining which of these techniques can be applied for clinical use. The unseeded biomatrix has the advantage of being available to surgeons at any time of a reconstructive operation. The development of a readymade bioactive biomatrix with predefined characteristics promoting tissue growth of the bladder would significantly improve the reconstructive possibilities of the urologist. Different biodegradable biomatrices have been used for preclinical studies. Amongst these, SIS, an acellular collagen matrix harvested from porcine small intestine, is one of the most thoroughly studied biomaterial used for the unseeded grafts. Bladder reconstruction with SIS has demonstrated regeneration of transitional epithelium, smooth muscle and nerves in various different animal models.[58,59] Although the initial results have been promising, long-term results have been poor. This is due to the complications such as fibrosis resulting in graft shrinkage, graft incrustation or infection.

Recently, Atala *et al*., published the first human clinical study with engineered bladders.[16] Seven patients with myelomeningocele and resultant dysfunctional bladders underwent bladder augmentations with autologous, smooth muscle and urothelial cell seeded scaffolds. These scaffolds were made of biodegradable collagen and polyglycolic acid composite. The engineered tissue improved overall bladder function in all patients, with mean follow-up of almost four years. All three patients who had composite scaffolds and omental wrap had better outcomes than earlier decellularized bladder submucosa scaffold cystoplasties. It seems appropriate to conclude that the omental wrap (vascular supply) had an important role in the success of the engineered bladders in the study by Atala *et al*. This aforementioned report is a milestone in tissue engineering of the bladder, but further studies and improvements are needed for widespread clinical implementation.

## CONCLUSION

Gastrointestinal tissue used for enterocystoplasty is associated with different short and long-term complications. Reservoir perforation must be considered in patients with acute abdominal pain or peritonitis and long-term follow-up of renal function is needed. In addition, one has to be aware of the long-term risk of malignancy in these procedures which has come to the attention of the urologists recently. Regarding these complications, there is a current necessity to develop alternative tissues for bladder augmentation by the help of tissue engineering protocols that will replace the integration of bowel segments into the urinary tract. Until then, intestinal cystoplasty still seems to be the gold standard due to the lack of promising alternative options.
